# Targeting IGF1R signaling enhances the sensitivity of cisplatin by inhibiting proline and arginine metabolism in oesophageal squamous cell carcinoma under hypoxia

**DOI:** 10.1186/s13046-023-02623-2

**Published:** 2023-03-28

**Authors:** Kang Fang, Mingchuang Sun, Zhuyun Leng, Yuan Chu, Ziying Zhao, Zhaoxing Li, Yunwei Zhang, Aiping Xu, Zehua Zhang, Li Zhang, Tao Chen, Meidong Xu

**Affiliations:** 1 Endoscopy Center, Department of Gastroenterology, Shanghai East Hospital, School of Medicine, Tongji University, Shanghai, 200120 China; 2 Department of Pathology, Shanghai East Hospital, School of Medicine, Tongji University, Shanghai, 200120 China

**Keywords:** Oesophageal squamous cell carcinoma, Cisplatin resistance, Proline and arginine metabolism, RTKs, Hypoxia

## Abstract

**Background:**

Cisplatin (DDP)-based chemotherapy is commonly adopted as the first-line treatment for patients with oesophageal squamous cell carcinoma (OSCC), but the high rate of drug resistance limits its clinical application and the underlying mechanisms at play remain unclear. The aims of this study were to elucidate the role of abnormal signal transmission and metabolism in the chemoresistance of OSCC under hypoxia and to identify targeted drugs that enhance the sensitivity of DDP chemotherapy.

**Methods:**

Upregulated genes in OSCC were determined by RNA sequencing (RNA-seq), the Cancer Genome Atlas (TCGA) database, immunohistochemistry (IHC), real-time quantitative PCR (RT-qPCR), and western blotting (WB). The clinicopathological significance of insulin-like growth factor-I receptor (IGF1R), argininosuccinate synthetase 1 (ASS1), and pyrroline-5-carboxylate reductase 1 (PYCR1) in OSCC was analysed using tissue micriarray (TMA). Metabolic abnormalities were determined by untargeted metabolomics analysis. The DDP-resistance role of IGF1R, ASS1, and PYCR1 in OSCC was investigated in vitro and in vivo.

**Results:**

Generally, tumour cells exist in a hypoxic microenvironment. By genomic profiling, we determined that IGF1R, as a receptor tyrosine kinase (RTK), was upregulated in OSCC under low-oxygen conditions. Clinically, enhanced IGF1R expression was associated with higher tumour stages and a poorer prognosis in OSCC patients, and its inhibitor, linsitinib, showed synergistic effects with DDP therapy in vivo and in vitro. Since oxygen-deprivation frequently lead to metabolic reprogramming, we further learned via metabolomics analysis that abnormal IGF1R pathways promoted the expression of metabolic enzymes ASS1 and PYCR1 by the transcriptional activity of c-MYC. In detail, enhanced expression of ASS1 promotes arginine metabolism for biological anabolism, whereas PYCR1 activates proline metabolism for redox balance, which maintains the proliferation ability of OSCC cells during DDP treatment under hypoxic conditions.

**Conclusion:**

Enhanced expression of ASS1 and PYCR1 via IGF1R pathways rewired arginine and proline metabolism, promoting DDP resistance in OSCC under hypoxia. Linsitinib targeting IGF1R signaling may lead to promising combination therapy options for OSCC patients with DDP resistance.

**Supplementary Information:**

The online version contains supplementary material available at 10.1186/s13046-023-02623-2.

## Background

OSCC, as the most common subtype of oesophageal cancer (84%), remains a leading cause of tumour mortality globally [1, 2]. At the regional level, OSCC patients in east Asia, especially in China, have experienced the heaviest disease burden [1]. In China, due to a lack of typical clinical symptoms, many patients are diagnosed in an advanced stage and have to receive DDP-based chemotherapy [3]. However, DDP is often restricted by innate and adaptive resistance [4]; only 19%–58% of patients receiving DDP-based postoperative neoadjuvant chemotherapy for OSCC show a complete response [5], while the response rate for patients receiving DDP-based palliative chemotherapy was also just 35% to 45% [3]. Drug resistance can be caused by multiple factors, such as the activation of abnormal signaling pathways, epigenetic modifications, and metabolic reprogramming [6]. Thus far, the mechanism of DDP resistance to OSCC has not been fully elucidated, and guidance on combining drug therapies to enhance cell sensitivity remains inadequate.

As is known, OSCC cells, especially those in the core of solid tumours, remain in a relatively oxygen-deficient microenvironment because of the mismatch between the tumour growth rate and vascular supply [7, 8]. To survive in such harsh conditions, tumour cells tend to change their original metabolic patterns; for example, enhanced glycolysis replaces the tricarboxylic acid (TCA) cycle to produce energy [9]. Targeting glycolysis metabolism has been proven to be a novel approach to improve the sensitivity of OSCC cells to chemotherapeutic drugs [10]. Theoretically, in addition to enhanced glycolysis, hypoxia could also lead to TCA cycle and electron transport chain (ETC) dysfunction, which affects ATP generation, redox homeostasis, synthesis of biomolecules, etc. [11]. However, a comprehensive view of the metabolic mechanisms at play in the DDP-chemoresistance of OSCC cells under hypoxia remains to be introduced.

In several recent studies, dysregulated RTK family members were found in OSCC patients [12, 13]. As ‘stress-receptors’, they can receive and transmit extracellular ‘harsh’ messages such as warnings about low oxygen levels to the cytoplasm, and initiate the intracellular downstream signal cascade to promote the growth, invasion, metabolism, and resistance phenotype of tumour cells [14]. Targeting RTKs, such as by using well-known tyrosine kinase inhibitors (TKIs), is a promising strategy for tumour therapy [15]. However, single-target drug resistance is prominent in practice, and few targeted RTK drugs have been approved for treating OSCC. Therefore, understanding the regulatory mechanism of RTKs for downstream pathways can help us to find new synergistic drugs.

In this study, we found that IGF1R, as an RTK, is significantly elevated in OSCC under oxyen-poor conditions. By integrating metabolomic and genomic profiling, we determined that IGF1R promotes the expression of the metabolic enzymes PYCR1/ASS1 via canonical RTK pathways. Enhanced expression of these two key enzymes activates arginine/proline metabolism, maintaining the redox balance and providing precursors for biological anabolism. Eventually, abnormal signal transmission combined with metabolic reprogramming leads to DDP resistance. Specifically, the IGF1R inhibitor linsitinib demonstrated synergistic effects with DDP therapy. Our study suggests that targeting IGF1R pathways and related metabolic alterations may improve the therapeutic effect of DDP in OSCC, which may be a new strategy for more efficient DDP-based chemotherapy.

## Materials and methods

### Cell culture, cell transfection, and small chemical inhibitors

The OSCC cell lines were maintained in DMEM supplemented with 10% foetal bovine serum at 37 °C in a humidified 5% CO_2_ atmosphere. Cell lines were provided by the Institute of Biochemistry and Cell Biology of the CAS (Shanghai, China). Hypoxic conditions were established using airtight chambers saturated with 95% N_2_ and 5% CO_2_, and two cell lines were grown at 1% O_2_ [16].

SiRNA-cMYC/JAK1/ERK1/IGF1R was purchased from GenePharma (Shanghai, China), and Lipofectamine 3000 (Invitrogen, Carlsbad, CA, USA) was used for cell transfection. We also purchased LV-Control and LV-shIGF1R/ASS1/PYCR1 lentivirus from Genechem Biotechnology (Shanghai, China) and selected the stable gene-expression cell lines with puromycin.

DDP was obtained from Sigma-Aldrich (St. Louis, MO, USA), and linsitinib was obtained from MedChemExpress (Monmouth Junction, NJ, USA). The combination index (CI) values between linsitinib and DDP at different dose–effect levels were calculated using the CompuSyn software (version 1.0; ComboSyn Inc., Paramus, NJ, USA) [17].

### Cell proliferation

To evaluate the proliferation of OSCC cells, CCK-8 assays (Dojindo Laboratories, Kumamoto, Japan) were performed. Briefly, a total of 2 × 10^3^ cells were seeded in 96-well plates and cultured for 0–96 h. Then, 10 μL of CCK-8 reagent and 90 μL of DMEM were added and the cells were incubated. After 1 h of incubation, the absorbance of cells at 450 nm was measured using a microplate reader.

### RT-qPCR assays, RNA-seq, and bioinformatic analysis

Total RNA was isolated using TRIzol reagent (Invitrogen, Carlsbad, CA, USA), following the manufacturer’s instructions. RNAs were transcribed into cDNAs using PrimeScript (DRR036A; Takara Bio, Tokyo, Japan). RT-qPCR was performed using SYBR Green (RR420A; Takara Bio). The primers are summarised in Supplementary Table S1. Expression levels were normalised to β-actin.

Library preparation for RNA-seq was conducted. Generally, 1 μg of high-quality RNA was used, and sequencing was carried out using HiSeq2500 (Illumina Inc., San Diego, CA, USA) at Genechem Biotechnology (Shanghai, China).

The RNA-seq data of OSCC tissues and paired normal tissues were obtained from TCGA database.

### WB analysis

Proteins were extracted from cultured cells, which was followed by immunoblotting with the primary antibodies. Protein concentrations were determined by Bradford assay. Proteins were separated by SDS-PAGE, transferred onto PVDF membranes (Millipore Corporation, Burlington, MA, USA), and probed with the indicated antibodies. Antibodies that recognise ASS1 (ab175607), PYCR1 (ab103314), IGF1R (ab182408), c-MYC (ab32072), c-MYC^T58^ (ab185655), JAK1 (ab133666), JAK1-P (ab138005), STAT3 (ab32500), STAT3-P (ab267373), PI3K (ab191606), PI3K-P (ab182651), AKT (ab179463), AKT-P (ab192623), mTORC (ab134903), and β-actin (ab8227) were purchased from Abcam (Cambridge, UK). Immunoblotting assays were performed as previously reported [18].

### Chromatin immunoprecipitation (ChIP) assay

ChIP assay was performed using an Upstate Biotechnology kit (Thermo Fisher Scientific, Waltham, MA, USA). Briefly, the cells were cross-linked with 1% formaldehyde plus 1.5 mmol/L of ethylene glycol-bis at room temperature. Cross-linked chromatin was sonicated and precipitated with antibodies against transcription factors. Quantitative real-time PCR was performed to measure the amount of bound DNA. The selected primers covering the c-MYC binding sites of the ASS1 and PYCR1 genes’ promoter regions were summarized in Supplementary Table S1.

### Luciferase assay

Fragments containing the ASS1 and PYCR1 promoter regions or mutants of the predictive c-MYC binding site were inserted upstream of the firefly luciferase coding sequences in the pGL3-basic reporter plasmid. OSCC cells were seeded in 96-well plates and then transiently transfected with pGL3-ctrl, pGL3-ASS1, and PYCR1 promoter; pGL3-ASS1 mut1 or pGL3-ASS1 mut2; pGL3-PYCR1 mut; and pTK-Renilla, with or without cDNA–c-MYC plasmid. The luciferase activities were determined using the Dual-Glo luciferase assay system (E2920; Promega Corporation, Madison, WI, USA).

### IHC and TMA

This study was approved by the institutional review board of Shanghai East Hospital, School of Medicine, Tongji University. IHC analysis was conducted as described previously [19]. A TMA consisting of tumour and para-cancerous tissue samples from 79 patients was created and subjected to IHC analysis. The results were scored by two pathologists who were blinded to patient information. In particular, the tissues of 25 patients pathologically diagnosed with early-stage cancer were further analzyed compared with para-neoplastic tissues.

### Metabolomics analysis

Transduced control or shRNA-ASS1/PYCR1 KYSE150 cells were cultured in low-oxygen medium for 24 h; then, untargeted metabolomics analysis was performed using a quantitative, automatic, and high-throughput LC–MS approach to simultaneously determine 324 metabolites belonging to different important primary metabolic pathways, such as glycolysis, aerobic respiration, TCA cycle, fatty acid oxidation (FAO), and gluconeogenesis [20]. The acquired raw data were analysed using XploreMET (version 3.0; Metabo-Profile, Shanghai, China).

### Measurement of NAD + and ATP level

The NAD + and ATP levels were determined using an NAD + /NADH quantification kit (Beyotime Biotechnology, Beijing, China), and an ATP assay kit (Beyotime) according to the manufacturers’ instructions, respectively.

### Animals

Four-week-old female nude mice were obtained from Vitalriver (Beijing, China), and 5.0 × 10^6^ cells were injected subcutaneously into the right flank of each mouse. To develop the primary cancer model, the mice were killed and tumours were removed after 4 weeks. To further test the sensitivity of OSCC to chemotherapeutic drugs, the mice were intraperitoneally injected with DDP for 4 weeks (2 mg/kg, twice per week). The tumour volume (mm^3^) of both models was calculated as 0.5 × length × width^2^. This in vivo study was approved by the animal care and use committee of Tongji University.

### Statistical analysis

Differences between groups were calculated using Student’s *t* test, one-way analysis of variance, Chi-squared test, or Fisher’s exact test. GraphPad Prism version 7.0 was used for all statistical analyses. All error bars show mean ± SEM. Significance is defined as *P < 0.05, **P < 0.01, ***P < 0.001.

## Results

### IGF1R was overexpressed in OSCC and associated with disease progression and poor prognosis

**Fig. 1:**
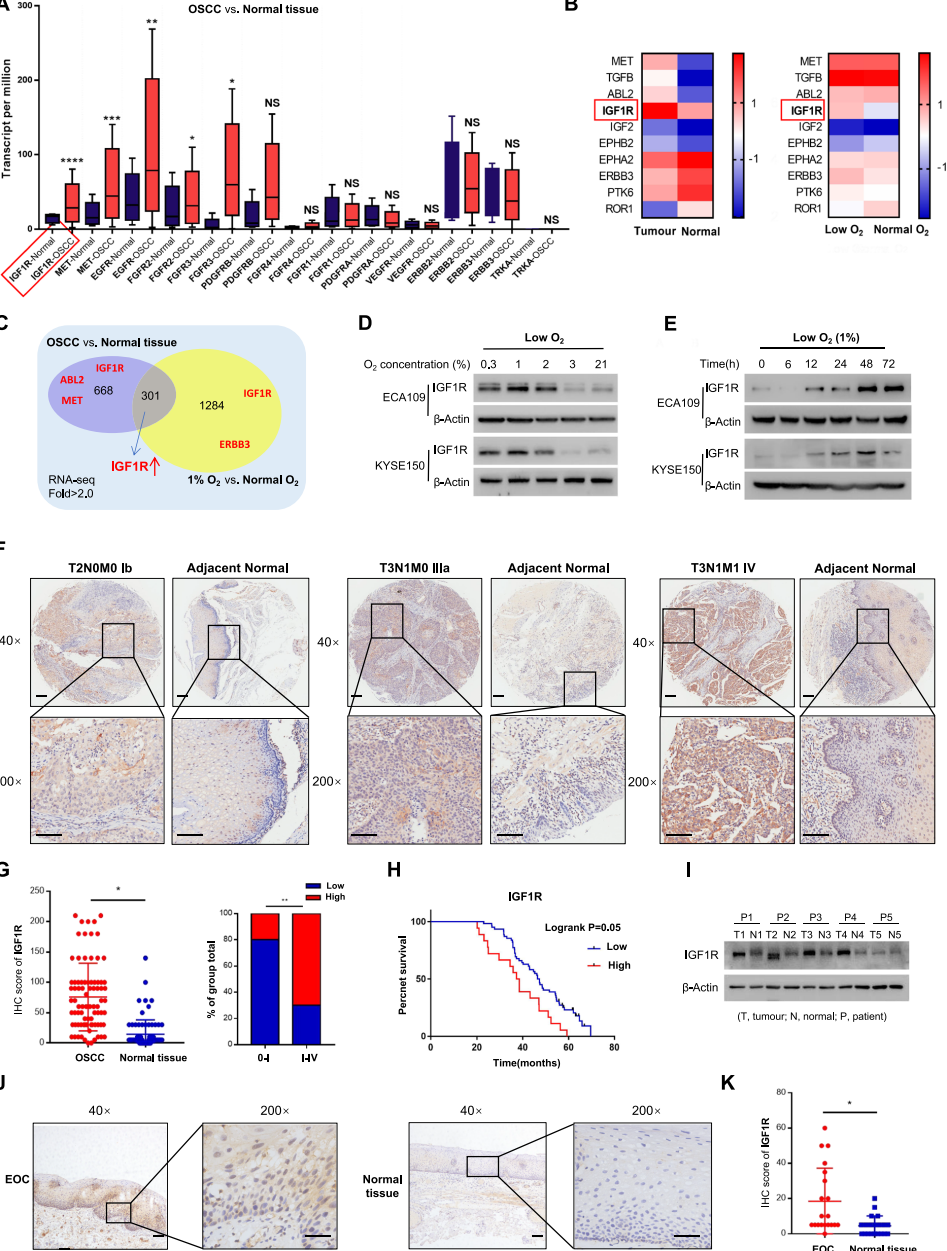
IGF1R was overexpressed in OSCC and associated with disease progression and poor prognosis. **A** Expression of common RTKs in OSCC tissues compared to adjacent normal tissues from RNA-seq data gathered from the TCGA. **B** A subset of abnormally expressed genes concerning RTKs in OSCC tissues (left) or in KYSE150 cells under limited-oxygen conditions (right) from RNA-seq data based on our specimens. **C** Venn diagram for crossovers of two subsets of genes in Fig. 1B. **D**, **E** Expression of IGF1R at protein levels in OSCC cell lines under different oxygen concentrations (D) and different times duration of hypoxia (E). **F** Representative TMA images of IGF1R protein in different OSCC stage specimens (Ib–IV) or adjacent normal tissue specimens. Scale bars represent 100 mm. **G** IHC scores of IGF1R in OSCC or normal tissues (left), Student’s *t* test; Percentage of high scores in 0–IV stage OSCC (right), Chi-squared test. **H** Correlation between the overall survival of OSCC patients and IGF1R expression. **I** Expression of IGF1R in OSCC tissues compared to adjacent normal tissues from representative clinical patients (*n* = 5). **J** Representative IHC images of IGF1R protein in EOC tissues or adjacent normal tissues. Scale bars represent 100 mm. **K** IHC scores of IGF1R in EOC tissues compared to normal tissues. NS: not signifcant, *p < 0.05, **p < 0.01, ***p < 0.001

As mentioned above, to investigate alterations in RTKs (common RTKs are listed in Table 1) related gene amplification in OSCC, we first analysed data from the TCGA. Among them, the expression levels of IGF1R, MET, EGFR, FGFR2, FGFR3, and PDGFRB were markedly upregulated in OSCCs compared to non-cancerous tissues, with the difference in IGF1R expression being the most statistically significant (Fig. 1A). Meanwhile, overexpression of MET and IGF1R was also found in our OSCC patient tissues via RNA-seq (Fig. 1B Left). To further analyse the role of dysregulated RTKs on chemoresistance under simulated conditions of limited oxygen (1%), we initially considered the selection of OSCC cell lines that had the greatest resistance to DDP. Recent findings have indicated that KYSE150 cells have the lowest drug sensitivity to DDP [21]. We thus examined the IC_50_ of DDP in different cell lines under low-oxygen conditions and found that this was indeed the case (Supplementary Fig. S1A). KYSE150 and ECA109 cells with the highest IC_50_ values were selected for subsequent RNA-seq (Fig. 1B Right). Consequently, only IGF1R was strikingly upregulated under hypoxia. By integrating RNA-seq of clinical tissue and cell line samples, we focused our subsequent research on IGF1R (Fig. 1C). Consistent with the RNA-seq results above, only IGF1R among the RTKs (other RTKs: Supplementary Fig. S1C-D), showed sustained elevated expression levels in a concentration- (Fig. 1D) and time- (Fig. 1E, Supplementary Fig. S1B) dependent manner under oxygen deprived conditions.

**Table 1:** Classification of common RTKs

Family	Members
EGFR	EGFR, ERBB2, ERBB3
IGFR	IGF1R
PDGFR	PDGFRα, PDGFRβ
VEGFR	VEGFR1, VEGFR2, VEGFR3
FGFR	FGFR1, FGFR2, FGFR3, FGFR4
NGFR	TRKA
HGFR	MET
EPHR	EPHA2, EPHB2
ROR	ROR1

*EGFR* Epidermal growth factor receptor, *PDGFR* Platelet-derived growth factor receptor, *VEGFR* Vascular endothelial growth factor receptor, *FGFR* Fibroblast growth factor receptor, *NGFR* Nerve growth factor receptor, *HGFR* Hepatocyte growth factor receptor, *EPHR* Ephrin receptor, *ROR* Receptor orphan

Clinically, the differences above were reflected by the results of TMA (Fig. 1F-H) and WB (Fig. 1I) analysis, where upregulated protein expression of IGF1R was found in tumour tissues compared to para-neoplastic tissues. Moreover, a high IGF1R expression level was positively associated with tumour stage (Fig. 1F-G, Table 2) and a poor prognosis for overall survival in 79 patients with OSCC (Fig. 1H, Supplementary Table S2). Specifically, a moderately elevated IGF1R protein abundance was found in early-stage oesophageal cancer (EOC, i.e., OSCC limited to the mucosa or superficial submucosa [22]) tissues compared to adjacent normal tissues (Fig. 1J-K), which could provide implications for early diagnosis and treatment. From the cellular viewpoint, consistent with the DDP IC_50_ experiment, ECA109 and KYSE150 cells had significantly greater expressions of IGF1R than other OSCC cell lines and the Het-1A normal oesophageal epithelial cell line (Supplementary Fig. S1E-F). Taken together, these data demonstrate that IGF1R is overexpressed in human OSCC, which may have prognostic implications and be related to DDP resistance under hypoxia.

**Table 2:** Association of IGF1R expression with clinical characteristics

Clinical characteristics	IGF1R expression		
	High	Low	*P* value^**a**^
**Gender**			
Male	38	27	0.568
Female	7	7	
**Age, years**			
≤ 60	13	11	0.807
> 60	32	23	
**Tumor size**			
≤ 3 cm	17	15	0.646
> 3 cm	28	19	
**Metastasis**			
Negtive	27	30	**0.006**
Positive	18	4	
**Clinical stage**			
0-II	32	31	**0.046**
III-IV	13	3	

^**a**^Statistical significance is determined by the Fisher’s exact test

### Inhibition of IGF1R enhanced the sensitivity of OSCC to DDP in vivo and in vitro

**Fig. 2:**
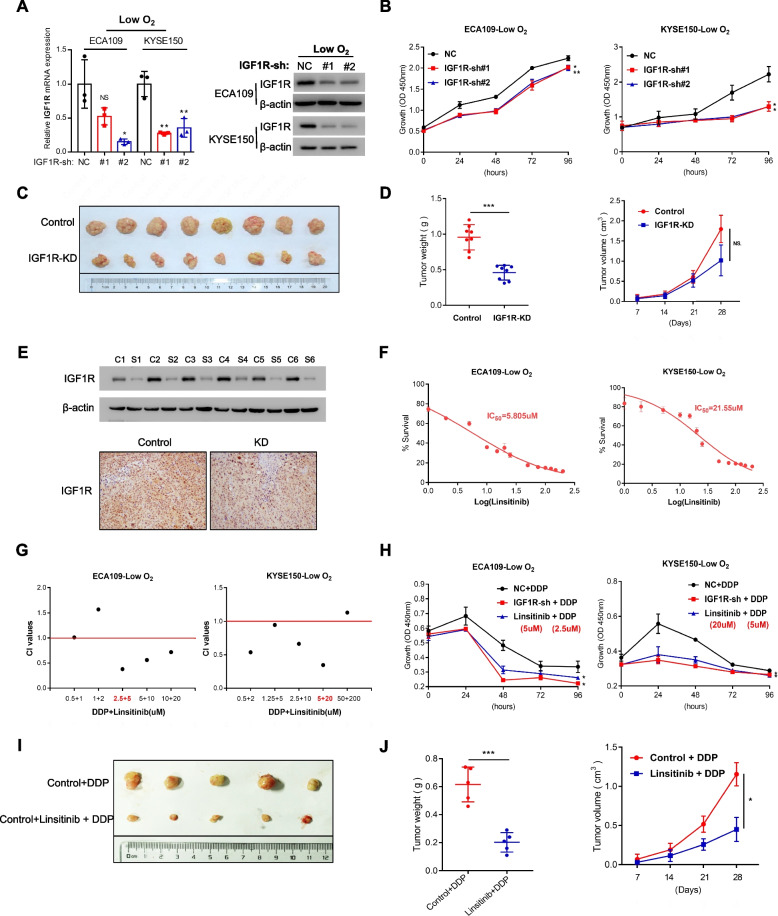
IGF1R inhibition enhanced the sensitivity of OSCC to DDP in vivo and in vitro. **A** Effect of shRNAs-IGF1R in ECA109 and KYSE150 cells at mRNA and protein levels under O_2_-poor mircoenvironment. **B** Proliferation ability was assessed by CCK-8 assay in OSCC cell lines transfected with shRNAs-control/IGF1R under hypoxia. **C** Representative images of tumours in a subcutaneous tumour model of nude mice injected with control/shIGF1R. **D** Weight (left) and volume (right) of subcutaneous tumour. *Abbreviation:* KD, knockdown. **E** Expression of IGF1R at the protein level in tumours of nude mice tested by WB and IHC. **F** IC_50_ of linsitinib in OSCC cell lines under hypoxic conditions. **G** The CI values of DDP at different concentrations combined with linsitinib in constant ratio, calculated by using the Chou–Talalay method. **H** Proliferation ability in OSCC cells treated with control/shIGF1R/linsitinib (linsitinib: ECA109, 5uM; KYSE150, 20uM) combined with DDP (ECA109, 2.5uM; KYSE150, 5uM). **I** Mice were intraperitoneally injected with DDP combined with linsitinib or not. Representative images of tumours from nude mice at day 28 are shown. **J** Weight (left) and volume (right) of subcutaneous tumour. Student’s *t* test. NS: not signifcant, *p < 0.05, **p < 0.01, ***p < 0.001

To explore the role of aberrantly expressed IGF1R on DDP resistance, we initially interfered with the expression of IGF1R (Fig. 2A), and a moderately inhibited proliferation ability of OSCC was found both in vitro (Fig. 2B) and in vivo (Fig. 2C-E). Furthermore, the IGF1R inhibitor linsitinib could effectively inhibit the expression of p-IGF1R (Supplementary Fig. S2A) at concentrations approximating to IC_50_ (Fig. 2F). Subsequently, linsitinib was employed for co-treatment with DDP (Figs. 2G, S2B), and their CI values at different dose–effect levels were all less than 1 (Table 3), which indicates that linsitinib in combination with DDP exerted synergistic effect to inhibit the proliferation of OSCC cells. These synergistic effects were also reflected in vitro (Fig. 2H) and in vivo (Fig. 2I-J). Overall, inhibited IGF1R expression may significantly increase the sensitivity of OSCC cells to DDP. Next, we would like to explore underlying mechanisms.

**Table 3:** CI values of DDP in combination with linsitinib in OSCC cells

Cell lines	CI values		
	ED50	ED75	ED90
ECA109	0.85	0.64	0.52
KYSE150	0.58	0.64	0.80

*ED50-90* Effective dose of 50–90% response

### The metabolic enzymes ASS1/PYCR1 were upregulated in OSCC under hypoxia

**Fig. 3:**
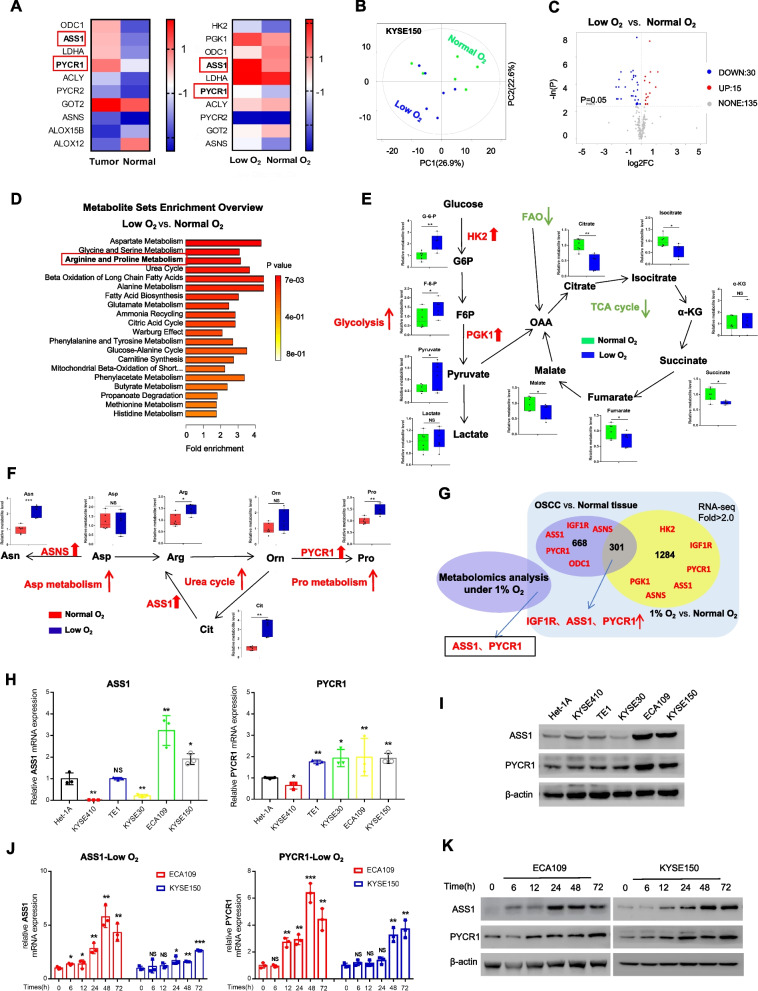
Metabolic enzymes ASS1 and PYCR1 were upregulated in OSCC. **A** Abnormally expressed genes regulating metabolic enzymes in OSCC tissues (left) compared to adjacent normal tissues, or in KYSE150 under low-oxygen conditions (right) compared to normal conditions using RNA-seq data from our specimens. **B** Principal component analysis (PCA) score plots obtained from the metabolite profiles of KYSE150 cells under normal or oxygen-deprived conditions. **C** Volcano map of the altered metabolites under hypoxia. **D** Enriched metabolic pathway analysis of abnormal metabolites under hypoxia. **E** Schematic representation of glycolysis and the TCA cycle. The relative levels of representative metabolites are shown (*n* = 6). *Abbreviations:* G6P, glucose 6-phosphate; F6P, fructose 6-phosphate; OAA, oxalacetic acid; HK2, hexokinase2; PGK1, phosphoglycerate kinase 1; α-KG, alpha-ketoglutaric acid. **F** Schematic representation of arginine/proline/aspartate metabolism (*n* = 6). *Abbreviations:* Asp, aspartate; Arg, arginine; Asn, asparagine; Glu, glutamate; Pro, proline; Orn, ornithine. **G** Venn diagram for crossovers between metabolomic (left) and genomic (right) profiling. **H**, **I** Expression of ASS1 and PYCR1 at mRNA (H) and protein (I) levels in different OSCC cell lines or the Het-1A. **J**, **K** Expression of ASS1 and PYCR1 at mRNA (J) and protein (K) levels in time-course experiments under limited oxygen conditions. Student’s *t* test. NS: not signifcant, *p < 0.05, **p < 0.01, ***p < 0.001

As previously described [23], metabolic adaptations contribute to cancer cell development in oxygen-poor microenvironments. Specifically, the alteration of metabolic enzymes is key to abnormalities of the overall metabolic pathway. Therefore, by integrating metabolomic and genomic profiling (Fig. 3G), we could expect to find activated metabolic enzymes under hypoxic conditions. On the one hand, both ASS1, which is the key enzyme in arginine metabolism, and PYCR1, which is responsible for proline synthesis, were highly expressed in both tumour tissues (Fig. 3A Left) and cell lines under limited oxygen conditions (Fig. 3A Right). On the other hand, untargeted metabolomics analysis was performed (Fig. 3B), and 45 abnormally accumulated metabolites were found in KYSE150 cells under hypoxia (Fig. 3C). By The Small Molecule Pathway Database analysis, these metabolites were found to be strongly related to multiple metabolism pathways (Fig. 3D and Supplementary Fig. S3A). Consistent with previous studies [24], we found that the TCA cycle and FAO were inhibited while accompanied by enhanced glycolysis (Fig. 3E), which may briefly indicate a homeostatic mechanism for ATP generation due to the lack of oxygen. By the way, fatty acid metabolism was suppressed in both lipid synthesis (Supplementary Fig. S3B) and carnitine metabolism (Supplementary Table S3), where carnitine metabolism involved in FAO. Of note, aspartate–arginine–proline metabolism was also enhanced (Fig. 3F). Our previous study demonstrated aberrant aspartate metabolism via the enzyme asparagine synthetase (ASNS) in OSCC [25]; therefore, we focused primarily on altered arginine/proline metabolism via ASS1 and PYCR1 (Fig. 3G).

### Altered expression of ASS1 and PYCR1 correlated with IGF1R

**Fig. 4:**
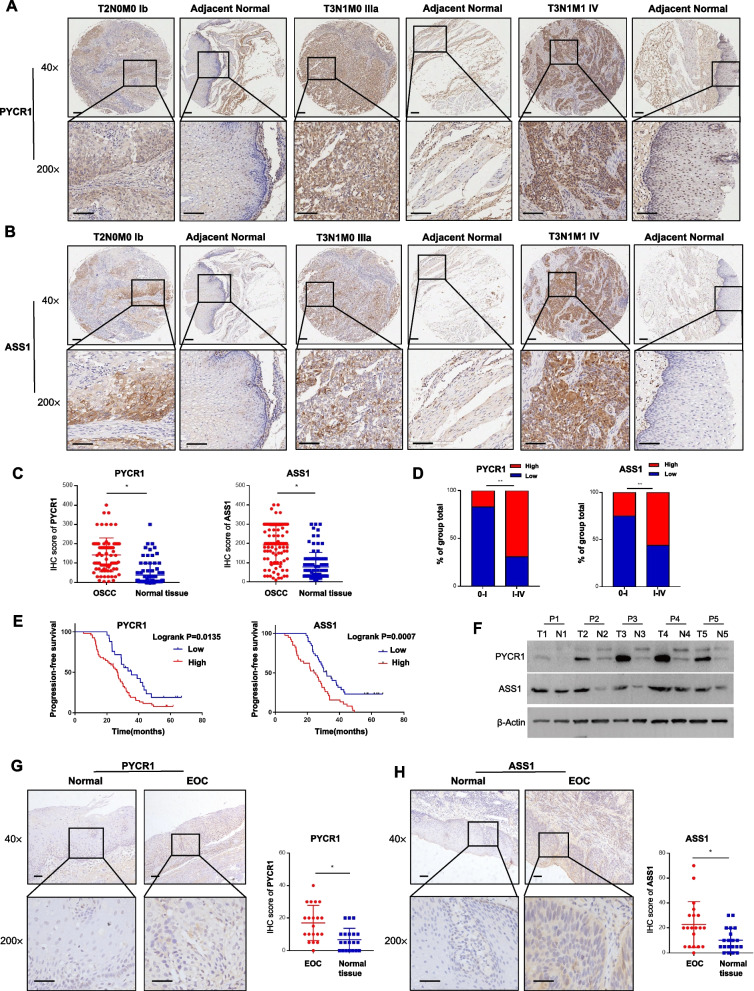
Altered expression of PYCR1 and ASS1 was associated with OSCC tumour progression and poor prognosis. **A**, **B** Representative TMA images of PYCR1 (A) and ASS1 (B) proteins in different OSCC stage specimens (Ib–IV) or normal tissue specimens. Scale bars represent 100 mm. **C** IHC scores of PYCR1 and ASS1 in OSCC or normal tissues, Student’s *t* test. **D** Percentage of high scores of PYCR1 and ASS1 in 0–IV stage OSCC, Chi-squared test. **E** Correlation between the progression-free survival of OSCC patients and PYCR1/ASS1 expression. **F** Expression of PYCR1 and ASS1 in OSCC compared to that in adjacent normal tissues from representative clinical patients (*n* = 5). **G**, **H** Representative IHC images of PYCR1 (G) and ASS1 (H) proteins in EOC or normal tissues. Scale bars represent 100 mm. *p < 0.05, **p < 0.01, ***p < 0.001

**Fig. 5:**
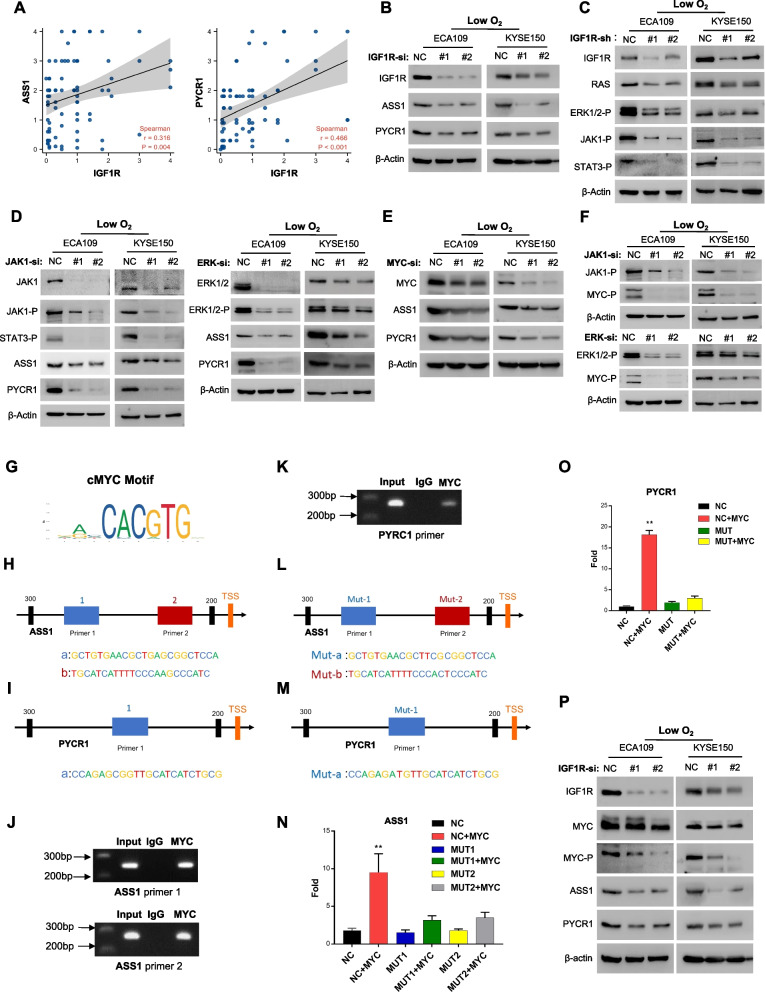
IGF1R regulates the expression of ASS1 and PYCR1 through its signaling pathway–c-MYC axis under hypoxia. **A** Correlation between IGF1R expression and PYCR1 or ASS1 expression in OSCC tissues obtained from TMA analysis. **B** Protein levels of ASS1 and PYCR1 in OSCC cells transfected with siRNA-control/IGF1R cultured under limited-oxygen conditions. **C** Protein levels of RAS/MAPK and JAK/STAT in OSCC cells transfected with shRNA-control/IGF1R. **D** Protein levels of ASS1 and PYCR1 in OSCC cells transfected with siRNA-control/JAK (left) or siRNA-control/ERK (right). **E** Protein levels of ASS1 and PYCR1 in OSCC cells transfected with siRNA-control/c-MYC. **F** Protein levels of c-MYC^T58^ in OSCC cells transfected with siRNA-control/JAK (Top) or siRNA-control/ERK (Bottom). **G**, **H**, **I** Predicted sequences of c-MYC motifs (G) at the ASS1 (H) and PYCR1 (I) promoters determined using JASPAR. **J**, **K** ChIP-PCR analysis of c-MYC enrichment at the ASS1 (J) and PYCR1 (K) promoters. **L**, **M** Schematic diagram of luciferase reporter plasmids, which were constructed containing the wild ASS1(L)/PYCR1(M) promoters or mutant c-MYC motif. **N**, **O** Transactivation activity of indicated ASS1 (N) and PYCR1 (O) promoters in KYSE150 cells from luciferase reporter gene assays. **P** Protein levels of cMYC, c-MYC.^T58^, ASS1 and PYCR1 in OSCC cells transfected with siRNA-control/IGF1R cultured in limited oxygen. Student’s *t* test. *p < 0.05, **p < 0.01, ***p < 0.001

We initially verified the expression levels of ASS1 and PYCR1 in OSCC. Consistent with IGF1R, both ASS1 and PYCR1 were most highly expressed in KYSE150 and ECA109 cell lines (Fig. 3H-I). In addition, hypoxia also stimulated the expression of both enzymes in a time-dependent manner (Fig. 3J-K). Moreover, TMA (Fig. 4A-D) and WB (Fig. 4F) revealed upregulated protein expression of ASS1/PYCR1 in OSCC patient tissues compared to para-neoplastic tissues. Clinically, the high ASS1/PYCR1 expression was associated with the tumour stage (Fig. 4A-D), even in EOC compared to normal tissue samples (Fig. 4G-H), and the progression-free survival of OSCC patients (Fig. 4E, Supplementary Fig. S4A). Furthermore, we found that both ASS1 and PYCR1 expression positively correlated with IGF1R in TMA (Fig. 5A). We therefore wondered whether ASS1 and PYCR1 are regulated by IGF1R.

### IGF1R regulates the expression of ASS1 and PYCR1 by related signaling pathways—cMYC axis

To further explore the mechanism by which IGF1R regulates ASS1 and PYCR1 expression, we first inhibited the expression of IGF1R and found that ASS1/PYCR1 was downregulated in vivo (Supplementary Fig. S5A) and in vitro (Fig. 5B), respectively. Previously, IGF1R had been proven to be responsible for phosphorylating downstream cascade proteins in two main signaling pathways (i.e., the PI3K/AKT and Ras/MAPK pathways) [26] that promote multiple cell functions. Thus, we inhibited IGF1R expression to examine various signaling pathways and found significant reductions in the phosphorylation levels in the Ras/MAPK pathway, as expected, and also in the JAK/STAT pathway (Fig. 5C) rather than in the PI3K/AKT pathway (Supplementary Fig. S5B). Furthermore, the downregulation of JAK and ERK1/2 was accompanied by decreased ASS1/PYCR1 expression (Fig. 5D), confirming that IGF1R regulates ASS1/PYCR1 via these two signaling pathways. To further identify the bridge between IGF1R pathways and ASS1/PYCR1, multiple transcription factors (TFs) have attracted our attention, which play a crucial role in regulating cancer metabolism during hypoxia [27, 28], and there transcriptional activity could be enhanced due to phosphorylation by RTK pathways [29]. As a result, among several common TFs (Supplementary Fig. S5C), only phosphorylated c-MYC showed clear regulatory relationships with ASS1/PYCR1 (Fig. 5E) and IGF1R pathways (Fig. 5F). To determine the direct regulation between c-MYC and ASS1/PYCR1, we performed JASPAR analysis (Fig. 5G), which indicated that both ASS1 (Fig. 5H) and PYCR1 (Fig. 5I) have c-MYC binding sequences in promoter regions. ChIP assay of c-MYC, followed by quantitative PCR, confirmed that c-MYC is directly bound to the ASS1 (Fig. 5J) and PYCR1 (Fig. 5K) promoters. In addition, dual-luciferase reporter assays revealed that c-MYC could promote the transcriptional activation of ASS1 (Fig. 5L, N) and PYCR1 (Fig. 5M, O). Taken together, these results suggest that altered expression of ASS1 and PYCR1 under hypoxic conditions is induced by the IGF1R signaling pathway–c-MYC axis (Fig. 5P).

### IGF1R reduces the sensitivity of OSCC cells to DDP by targeting arginine metabolism

**Fig. 6:**
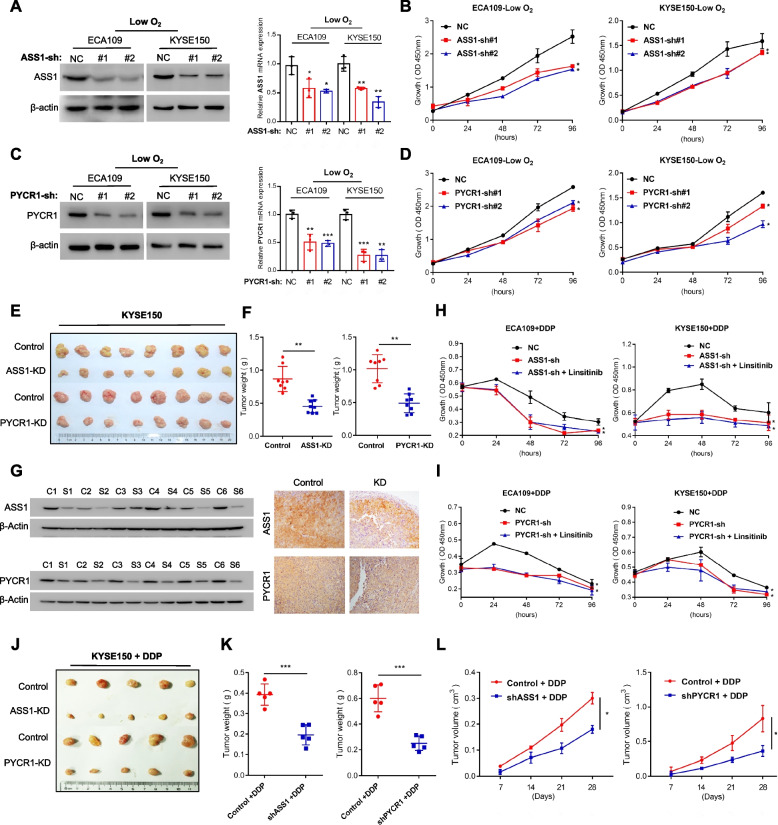
The role of ASS1 and PYCR1 in OSCC. **A**, **C** Effects of shRNA-ASS1 (A) or shRNA-PYCR1 (C) in ECA109 and KYSE150 cells under low-O_2_ mircoenvironment. **B**, **D** Proliferation ability in OSCC cells transfected with control/shASS1 (B) and control/shPYCR1 (D). **E–G** Representative images of tumours in nude mice injected by control/shASS1/shPYCR1 (E). Weight (F) of subcutaneous tumour size. Protein levels of ASS1 and PYCR1 in control or shRNA-ASS1/PYCR1 xenografted tumours of nude mice (G). C = shRNA-Control sample; S = shRNA-ASS1/PYCR1 sample. **H**, **I** Proliferation ability was assessed in OSCC cells divided into groups control + DDP, shASS1(H)/shPYCR1(I) + DDP, shASS1(H)/shPYCR1(I) + Linsitinib + DDP. **J**, **K**, **L** Representative images of tumours in nude mice injected by control/shASS1/shPYCR1 combind with DDP (J). Weight (K) and volume (L) of subcutaneous tumour size. Student’s *t* test. *p < 0.05, **p < 0.01, ***p < 0.001

**Fig. 7:**
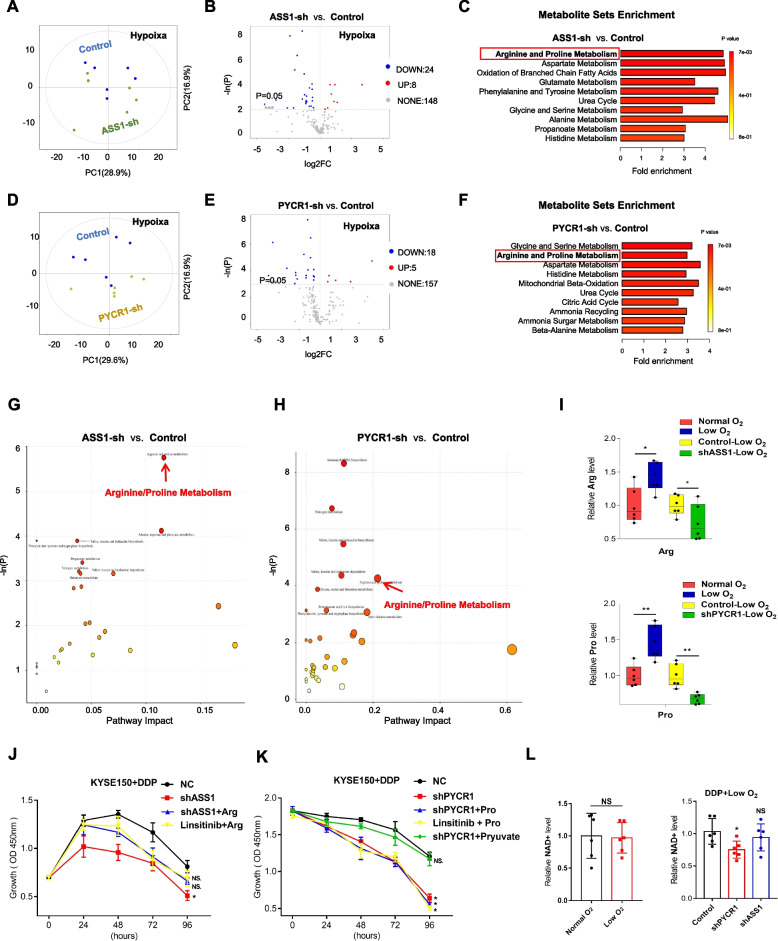
IGF1R reduces the sensitivity of OSCC cells to DDP by targeting arginine and proline metabolism under hypoxia. **A**, **D** PCA score plots obtained from metabolite profiles of KYSE150 transfected with control and shASS1(A)/PYCR1(D). **B**, **E** Volcano map of the altered metabolites in group shASS1(B)/PYCR1(E) compared to group control. **C**, **F** Enriched metabolic pathways analysis of abnormal metabolites in group shASS1(C)/PYCR1(F) compared to group control. **G**, **H** Bubble diagram of enrichment analysis on abnormal metabolites in group shASS1(G)/PYCR1(H) compared to group control. **I** The relative arginine (Top) and proline (Bottom) levels in KYSE150 in different groups. **J**, **K** Proliferation ability in different groups treated with DDP under low oxygen conditions. **L** The relative NAD + levels in low oxygen conditions compared to normal conditions (left), and in groups shPYCR1 + DDP, shASS1 + DDP compared to groups control + DDP (right). Student’s *t* test. NS: not signifcant, *p < 0.05, **p < 0.01

To further investigate the role of enhanced ASS1 and PYCR1 in DDP resistance, we initially interfered with the expression of ASS1 (Fig. 6A) and PYCR1 (Fig. 6C), observing a slightly inhibited proliferation ability in vitro (Fig. 6B, D) and in vivo (Fig. 6E-G, Supplementary Fig. S6A), respectively. Consistent with the effect of the IGF1R inhibitor linsitinib, shASS1/PYCR1 demonstrated a synergistic effect on tumour-proliferation inhibition with DDP in vitro (Fig. 6H-I) and in vivo (Fig. 6J-L). Next, metabolomics analysis was also conducted to investigate the role of ASS1 (Fig. 7A) and PYCR1 (Fig. 7D) in metabolic reprogramming. Abnormalities induced by ASS1 (Fig. 7B, C, G) and PYCR1 (Fig. 7E, F, H) inhibition were strongly related to arginine/proline metabolism. More specifically, proline levels were reduced in shRNA-PYCR1 cells, as well as arginine in shRNA-ASS1 cells (Fig. 7I). Further, to uncover the role of arginine, we performed rescue experiments. Compared to the control + DDP group, the suppressed proliferation capacity in the shASS1 + DDP or linsitinib + DDP group was partially rescued by supplementation with exogenous arginine (Fig. 7J). These data suggest that IGF1R promotes arginine metabolism via regulating ASS1 under hypoxia, leading to arginine accumulation, which serves as a biosynthetic precursor to maintain the proliferation ability in the chemotherapy period [30].

### IGF1R reduces the sensitivity of OSCC cells to DDP by targeting proline metabolism

In contrast to arginine, exogenous proline failed to rescue proliferation in the shPYCR1 + DDP or linsitinib + DDP group (Fig. 7K). Therefore, rather than proline accumulation, enhanced proline metabolism may promote DDP-resistance in tumour cells in other ways. Given the enhanced glycolysis and inhibited TCA cycle in shPYCR1 cells compared to control cells under hypoxia (Supplementary Fig. S7A), we detected the ATP level to confirm whether enhanced PYCR1 expression was involved in the ATP balance, which might influence the proliferation ability. Consequently, neither low oxygen nor ASS1/PYCR1 inhibition (Supplementary Fig. S7B) obviously affects ATP levels.

As is known, proline synthesis catalysed by PYCR1 is accompanied by NAD + biosynthesis, which plays a key role in maintaining the redox balance [31]. Theoretically, impairment of the TCA cycle caused by low oxygen not only leads to a reduction in ATP generation but also to a decrease in the accumulation of multiple intermediate metabolites in the TCA cycle [32], inhibiting the synthesis of anabolic precursors and ultimately cell proliferation. Under hypoxia, the decrease in NAD + levels caused by damage to the ETC may be a reason for the repressed metabolite accumulation from the TCA cycle. We therefore wondered whether NAD + from enhanced proline metabolism via PYCR1 compensated for the TCA cycle. As expected, attenuated PYCR1 expression suppressed the NAD + level (Fig. 7L). Finally, we added additional exogenous pyruvate in the DDP + shPYCR1 group, which could support intermediate metabolite accumulation in the TCA cycle; as a result, exogenous pyruvate reversed the inhibited proliferation caused by shPYCR1 + DDP treatment (Fig. 7K). Taken together, IGF1R promotes proline metabolism via regulating PYCR1, providing sufficient NAD + to maintain the redox balance in the TCA cycle, ultimately supporting cell proliferation in the chemotherapy period.

## Discussion

OSCC is a highly malignant tumour of the digestive system, even in its early stage. In China, frontline treatments for OSCC include operative treatment, chemo- and radiotherapy with or not surgical resection, but observations of poor prognosis and a low five-year survival rate persist [33]. Patients in advanced stages are mostly treated with combination therapy based on DDP or fluoropyrimidines [3, 34]; however, drug resistance and cytotoxicity limit their clinical application. As mentioned above, DDP resistance often occurs due to multiple carcinogenic factors, and targeting therapy to said factors is a promising option to alleviate chemoresistance by increasing treatment efficiency and reducing drug cytotoxicity. Regrettably, few targeting drugs have been approved for OSCC therapy at present. Therefore, it is urgent to explore the mechanisms involved in OSCC chemoresistance to find potential therapeutic targets.

Herein, we found that the abnormal IGF1R pathway is a potential target for OSCC therapy. Under low-oxygen conditions, IGF1R induces DDP resistance by enhancing ASS1 and PYCR1 expression to promote arginine and proline metabolism. Combining the IGF1R inhibitor linsitinib and DDP led to synergistic effects on inhibiting cell proliferation in vitro and in vivo.

Like other tumours, OSCC is exposed to limited-oxygen microenvironments during its rapid development. Though previous studies have partially confirmed how oncogenes help OSCC cells to tolerate limited oxygen conditions [35, 36], more work is necessary to grasp the overall landscape of the ‘metabolic adaptation’ to these ‘harsh’ conditions. In our study, enhanced expression of the enzymes ASS1 was found in OSCC cells under hypoxia. Intriguingly, the dual role of ASS1 has manifested in different cancer types; specifically, ASS1 is downregulated in breast cancer, hepatocellular carcinoma, etc. [4, 37–39]. Lacking ASS1 may promote the proliferation of these cancer cells by the conversion of aspartate to pyrimidine synthesis. Yet as a carcinogenic factor, ASS1 is found to be highly expressed in a few other tumours [40–42]. Nevertheless, the precise role of ASS1 in OSCC is rarely mentioned. In this study, we proved that upregulated ASS1 promoted arginine metabolism, leading to arginine accumulation, which facilitated biosynthesis and maintained the proliferation ability in the chemotherapy period.

PYCR1 catalyses the reduction of P5C to proline, and high expression of PYCR1 has been reported in other cancer types, including bladder cancer, gastric cancer, etc. [43–45]. However, the role of PYCR1 in OSCC has not been studied. We found that IGF1R promotes proline metabolism via regulating PYCR1, compensating the NAD + pool in the TCA cycle. Sufficient NAD + from proline metabolism maintained the redox balance and promoted intermediate metabolite accumulation in the TCA cycle, ultimately supporting cell proliferation in the chemotherapy period.

Accumulating evidence shows that RTKs and their related pathways are frequently dysregulated in OSCC [13, 46–48]. IGF1R, as a transmembrane glycoprotein and stress-receptor, could feel the stress outside and send these stress signals to intracellular, activating downstream pathways to regulate cell proliferation, migration, and chemotherapeutic resistance [14]. However, single-target RTKs have been proven to be inefficient due to multiple factors, such as the negative feedback activation in other oncogenic pathways [49] and the existence of complex crosstalks between different RTK pathways [12]. Combination therapy is emerging as a superior option; for example, the HER2 inhibitor trastuzumab deruxtecan, combined with DDP, has been approved as a first-line treatment in certain specific types of cancer [50]. Our studies demonstrated that abnormal activated IGF1R signaling pathways are associated with poor prognosis in OSCC patients. And the IGF1R inhibitor linsitinib enhanced the sensitivity of DDP chemotherapy. Activated arginine and proline metabolism via IGF1R may explain the mechanism of chemotherapy resistance.

## Conclusions

**Fig. 8:**
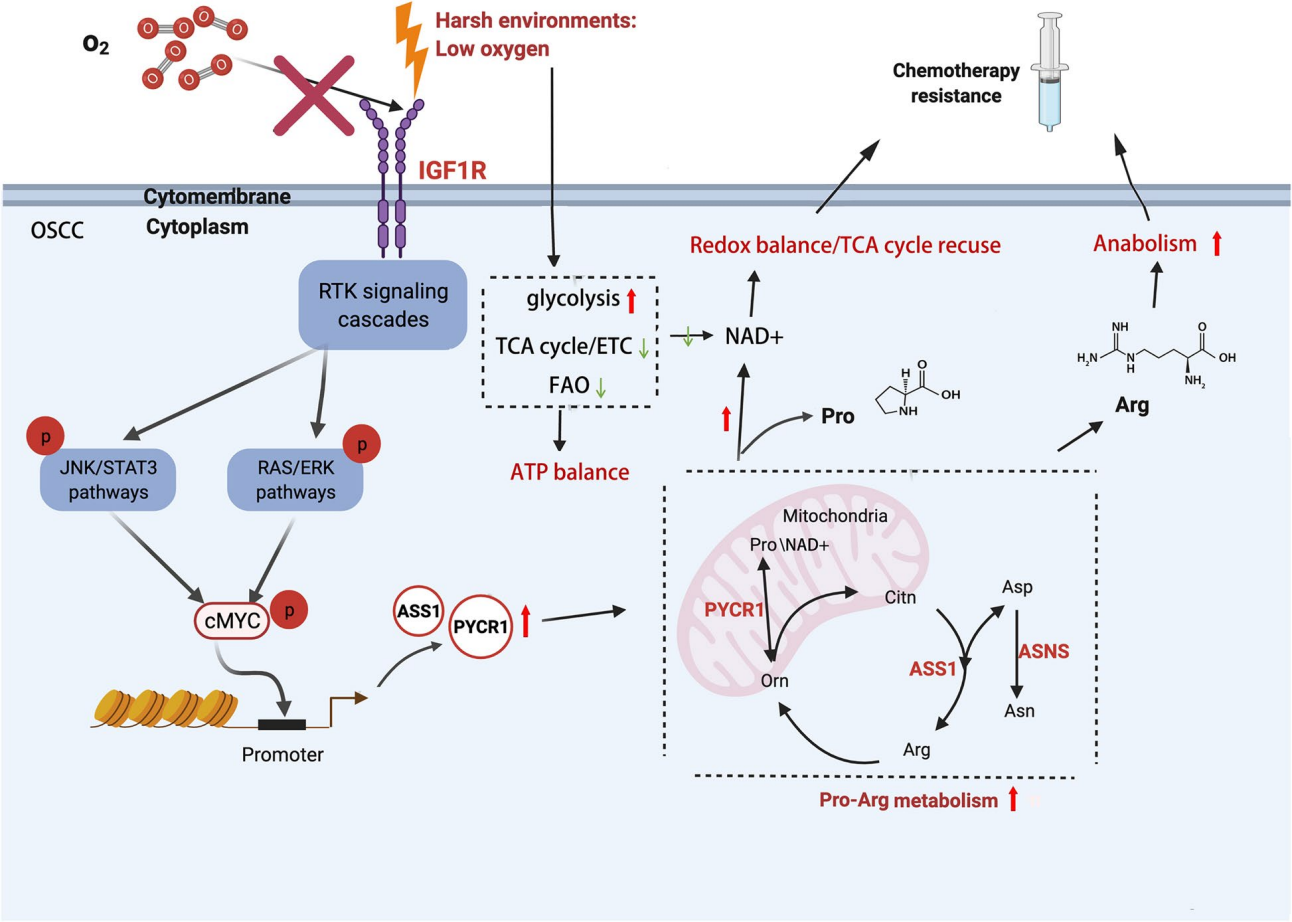
Graphical summary of DDP-resistance role of activated IGF1R pathways and arginine/proline metabolism in OSCC under limited-oxygen mircoenvironments

In summary, we found that activated IGF1R pathways play an important role in OSCC chemotherapy resistance under limited-oxygen mircoenvironments. Targeting IGF1R and related arginine/proline metabolism may be a key method to alleviate DDP resistance (Fig. 8). These findings improve our understanding of signal transmission and metabolic reprogramming in DDP resistance. Combination therapy with DDP and IGF1R inhibitors may be beneficial for OSCC treatment.

## Supplementary Information

**Additional file 1: Fig. S1.** A IC_50_ of DDP in ECA109 and KYSE150 under hypoxic conditions. B Expression of IGF1R at mRNA levels in time course experiments under hypoxia. C, D Expression of EGFR, MET, FGFR2, FGFR3 at mRNA (C) and protein (D) levels in time course experiments under conditions of limited oxygen. E, F Expression of IGF1R at mRNA (E) and protein (F) levels in different OSCC cell lines or the Het-1A oesophageal epithelial cell line. Student’s *t* test. NS: not signifcant, *p<0.05, **p<0.01, ***p<0.001. **Fig. S2.** A Effect of different concentrations of linsitinib on the inhibition of IGF1R/p-IGF1R expression. B Isobologram for Combo: effects of DDP and linsitinib combination on the inhibition of ECA109 and KYSE150 growth. **Fig. S3.** A Bubble diagram of enrichment analysis on abnormal metabolites in KYSE150s under low oxygen conditions compared to normal conditions. B The relative oleate and palmitoleate (representative metabolites in lipid synthesis metabolism) levels in KYSE150s under low oxygen conditions compared to normal conditions. **Fig. S4.** A Correlation between the overall survival of OSCC patients and PYCR1/ASS1 expression. **Fig. S5.** A Protein levels of IGF1R, ASS1 and PYCR1 in shRNA-control/IGF1R xenografted tumours of nude mice. C = shRNA-Control sample; S = shRNA-IGF1R sample. Right: Relative gray values of ASS1 and PYCR1 expression for all samples (*n *= 12) between group C and S. B Protein levels of PI3K/AKT in KYSE150 cells transfected with shRNA-Control/IGF1R cultured in limited oxygen. C Protein levels of ASS1 and PYCR1 in KYSE150 cells transfected with siRNA-Control or siRNA-NRF2 (left) /-ATF4 (right). Student’s *t* test. *p<0.05, **p<0.01. **Fig. S6.** A Volume of subcutaneous tumour size between group control and groups shASS1/shPYCR1. Student’s *t* test. NS: not signifcant. **Fig. S7.** A Schematic representation of glycolysis and TCA cycle under limited oxygen conditions. The relative levels of representative metabolites are shown (*n *= 6). Abbreviations are mentioned above. B The relative ATP levels in low oxygen conditions compared to normal conditions (left), and in groups shPYCR1+DDP, shASS1+DDP compared to groups control+DDP (right). Student’s *t* test. *p<0.05, **p<0.01.

**Additional file 2: Table S1.** Primers for real-time PCR assays.

**Additional file 3: Table S2.** Association of prognosis with clinical characteristics.

**Additional file 4: Table S3.** Summary of the carnitine metabolism.

## Data Availability

The datasets used and analyzed during the current study are available from the corresponding author on reasonable request.
