# Supplementary material for: Targeting IGF1R signaling enhances the sensitivity of cisplatin by inhibiting proline and arginine metabolism in oesophageal squamous cell carcinoma under hypoxia
Source: J Exp Clin Cancer Res. 2023 Mar 28;42:73. doi: 10.1186/s13046-023-02623-2 (PMC10044411; doi:10.1186/s13046-023-02623-2)
Supplement: Supplementary file 1 — Additional file 1: Fig. S1. A IC50 of DDP in ECA109 and KYSE150 under hypoxic conditions. B Expression of IGF1R at mRNA levels in time course experiments under hypoxia. C, D Expression of EGFR, MET, FGFR2, FGFR3 at mRNA (C) and protein (D) levels in time course experiments under conditions of limited oxygen. E, F Expression of IGF1R at mRNA (E) and protein (F) levels in different OSCC cell lines or the Het-1A oesophageal epithelial cell line. Student’s t test. NS: not signifcant, *p<0.05, **p<0.01, ***p<0.001. Fig. S2. A Effect of different concentrations of linsitinib on the inhibition of IGF1R/p-IGF1R expression. B Isobologram for Combo: effects of DDP and linsitinib combination on the inhibition of ECA109 and KYSE150 growth. Fig. S3. A Bubble diagram of enrichment analysis on abnormal metabolites in KYSE150s under low oxygen conditions compared to normal conditions. B The relative oleate and palmitoleate (representative metabolites in lipid synthesis metabolism) levels in KYSE150s under low oxygen conditions compared to normal conditions. Fig. S4. A Correlation between the overall survival of OSCC patients and PYCR1/ASS1 expression. Fig. S5. A Protein levels of IGF1R, ASS1 and PYCR1 in shRNA-control/IGF1R xenografted tumours of nude mice. C = shRNA-Control sample; S = shRNA-IGF1R sample. Right: Relative gray values of ASS1 and PYCR1 expression for all samples (n = 12) between group C and S. B Protein levels of PI3K/AKT in KYSE150 cells transfected with shRNA-Control/IGF1R cultured in limited oxygen. C Protein levels of ASS1 and PYCR1 in KYSE150 cells transfected with siRNA-Control or siRNA-NRF2 (left) /-ATF4 (right). Student’s t test. *p<0.05, **p<0.01. Fig. S6. A Volume of subcutaneous tumour size between group control and groups shASS1/shPYCR1. Student’s t test. NS: not signifcant. Fig. S7. A Schematic representation of glycolysis and TCA cycle under limited oxygen conditions. The relative levels of representative metabolites are shown (n = 6). Abbreviations ar [file 13046_2023_2623_MOESM1_ESM.pdf]

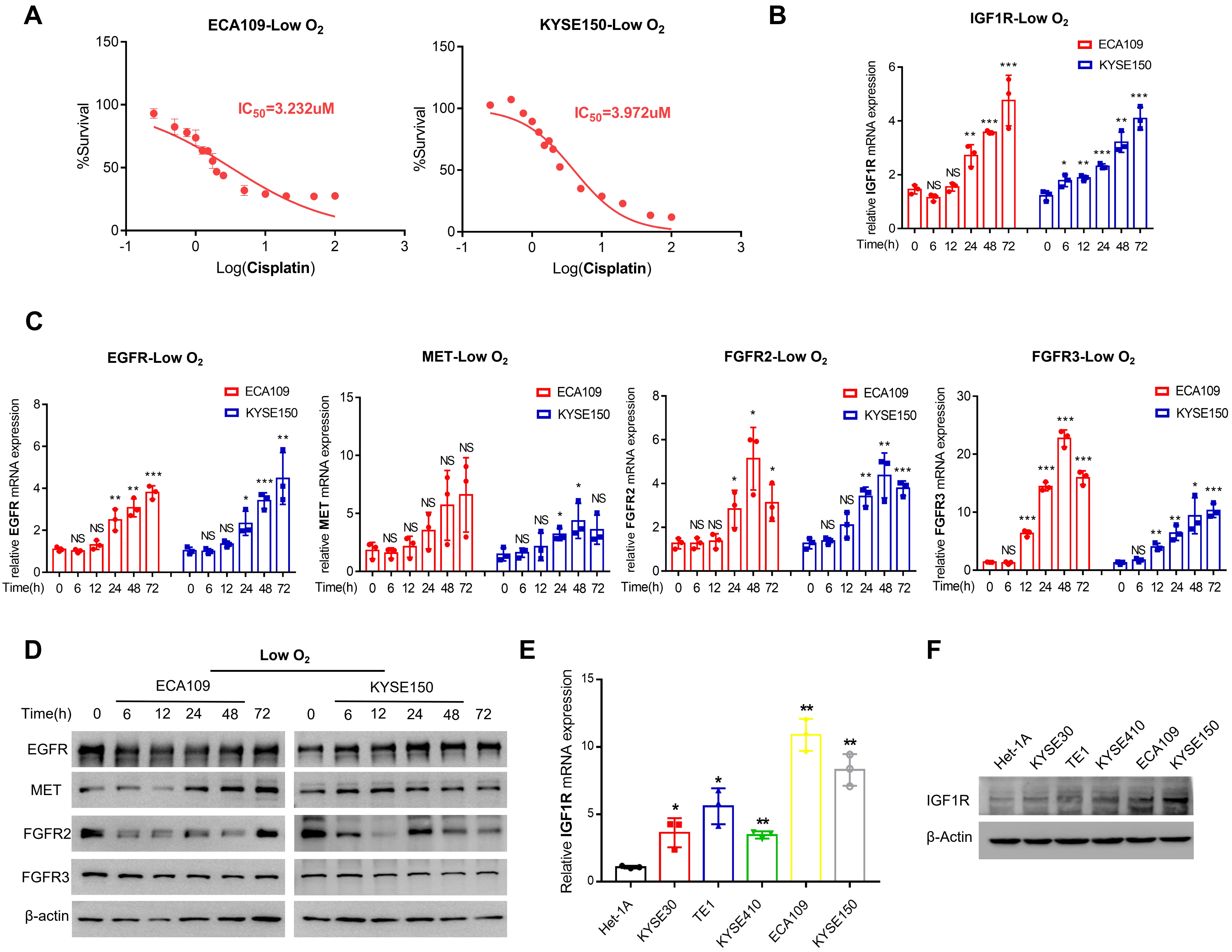
A
ECA109-Low O2
IC50=3.232uM
%Survival
Log(Cisplatin)
KYSE150-Low O2
IC50=3.972uM
%Survival
Log(Cisplatin)
B
IGF1R-Low O2
ECA109
KYSE150
relative IGF1R mRNA expression
Time(h) 0 6 12 24 48 72 0 6 12 24 48 72
C
EGFR-Low O2
relative EGFR mRNA expression
MET-Low O2
relative MET mRNA expression
FGFR2-Low O2
relative FGFR2 mRNA expression
FGFR3-Low O2
relative FGFR3 mRNA expression
ECA109
KYSE150
Time(h) 0 6 12 24 48 72 0 6 12 24 48 72
D
Low O2
ECA109
KYSE150
Time(h) 0 6 12 24 48 72 0 6 12 24 48 72
EGFR
MET
FGFR2
FGFR3
β-actin
E
Relative IGF1R mRNA expression
Het-1A
KYSE30
TE1
KYSE410
ECA109
KYSE150
F
Het-1A
KYSE30
TE1
KYSE410
ECA109
KYSE150
IGF1R
β-Actin

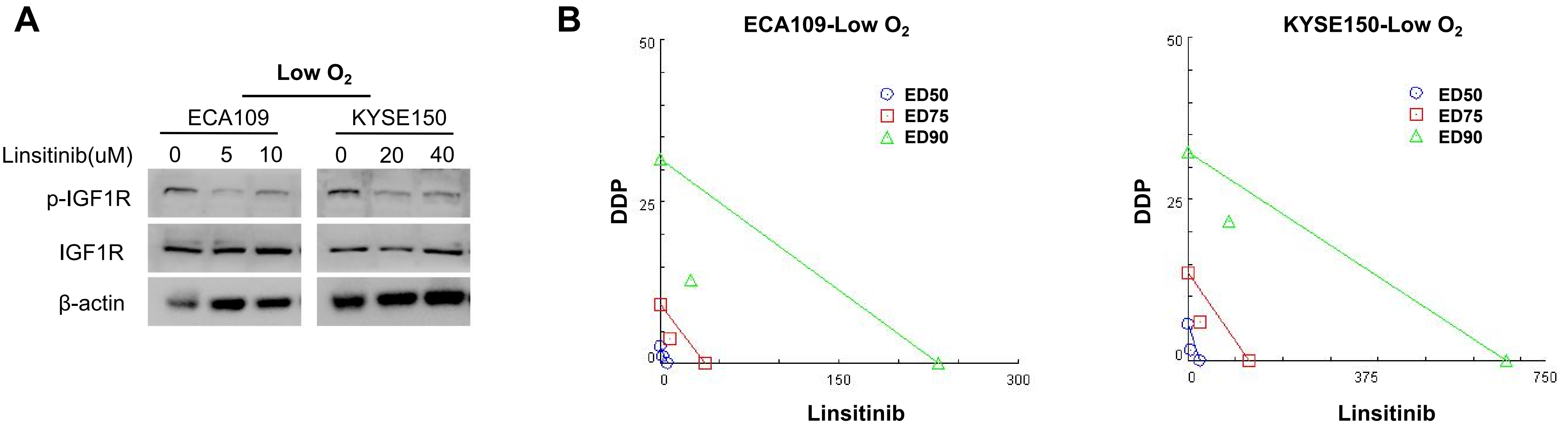
A
Low O_2
ECA109
KYSE150
Linsitinib(uM)
0
5
10
0
20
40
p-IGF1R
IGF1R
β-actin
B
ECA109-Low O_2
KYSE150-Low O_2
ED50
ED75
ED90
DDP
Linsitinib

A

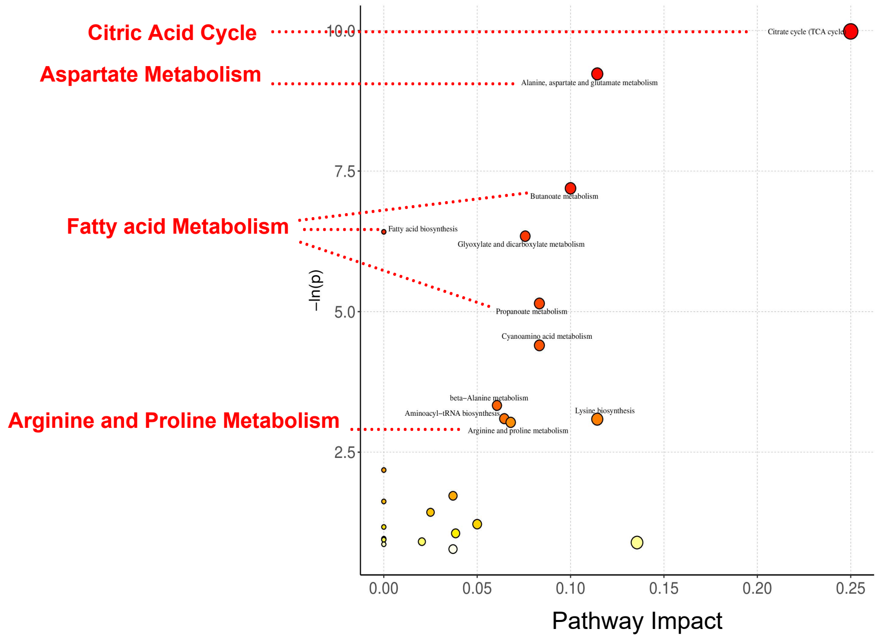

Citric Acid Cycle
Aspartate Metabolism
Fatty acid Metabolism
Arginine and Proline Metabolism
Citrate cycle (TCA cycle)
Alanine, aspartate and glutamate metabolism
Butanoate metabolism
Fatty acid biosynthesis
Glyoxylate and dicarboxylate metabolism
Propanoate metabolism
Cyanoamino acid metabolism
beta−Alanine metabolism
Aminoacyl−tRNA biosynthesis
Lysine biosynthesis
Arginine and proline metabolism
−ln(p)
10.0
7.5
5.0
2.5
0.00
0.05
0.10
0.15
0.20
0.25
Pathway Impact

B

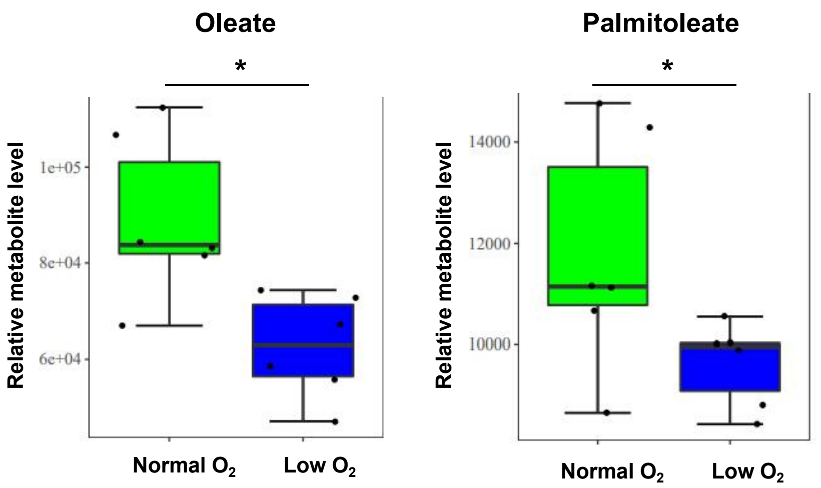

Oleate
*
Relative metabolite level
1e+05
8e+04
6e+04
Normal O_2
Low O_2
Palmitoleate
*
Relative metabolite level
14000
12000
10000
Normal O_2
Low O_2

A

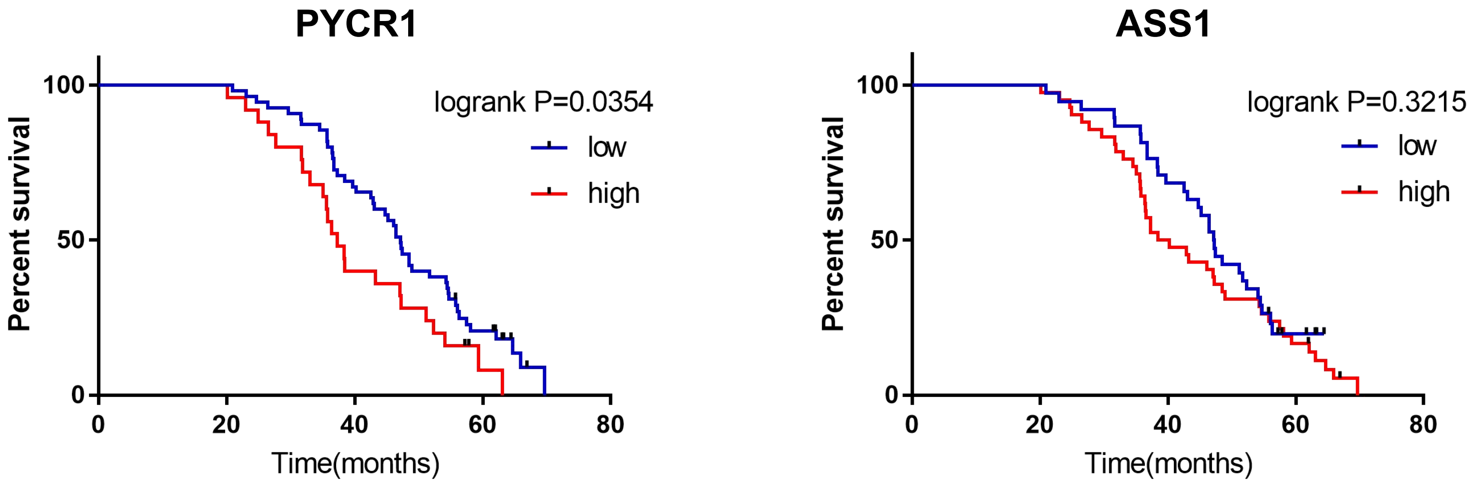

PYCR1
Percent survival
logrank P=0.0354
low
high
100
50
0
0
20
40
60
80
Time(months)
ASS1
Percent survival
logrank P=0.3215
low
high
100
50
0
0
20
40
60
80
Time(months)

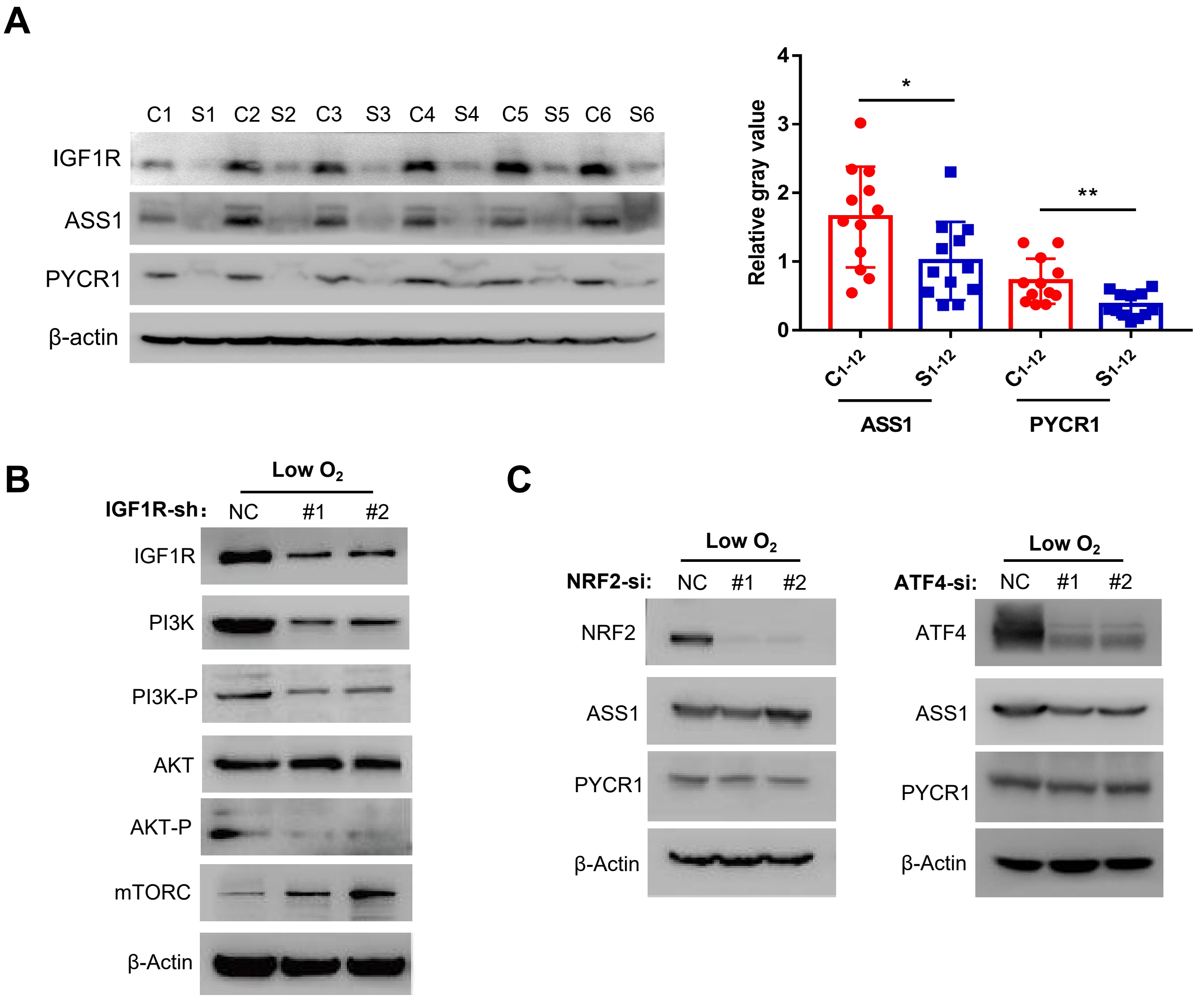
A
C1 S1 C2 S2 C3 S3 C4 S4 C5 S5 C6 S6
IGF1R
ASS1
PYCR1
β-actin
Relative gray value
0
1
2
3
4
*
**
C1-12
S1-12
C1-12
S1-12
ASS1
PYCR1
B
Low O2
IGF1R-sh:
NC
#1
#2
IGF1R
PI3K
PI3K-P
AKT
AKT-P
mTORC
β-Actin
C
Low O2
NRF2-si:
NC
#1
#2
NRF2
ASS1
PYCR1
β-Actin
Low O2
ATF4-si:
NC
#1
#2
ATF4
ASS1
PYCR1
β-Actin

A

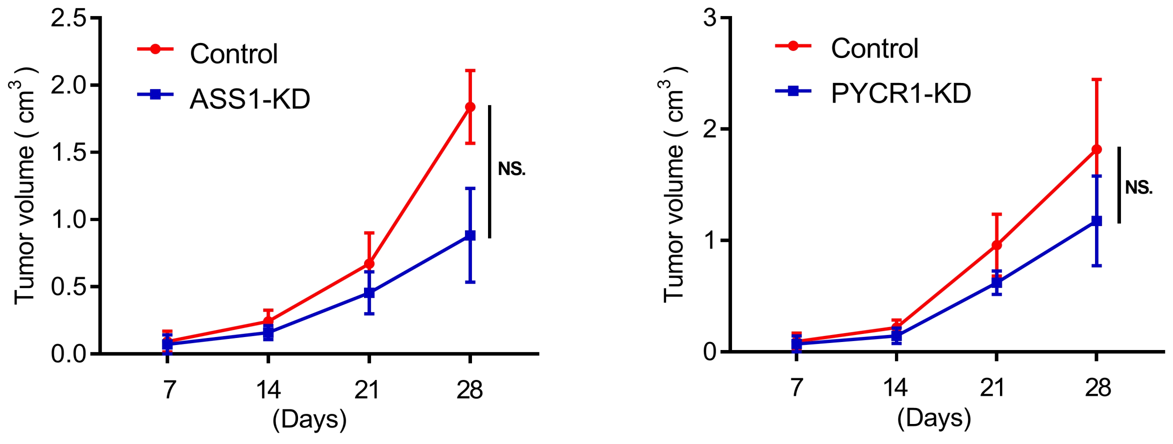

Tumor volume (cm3)
2.5
2.0
1.5
1.0
0.5
0.0
Control
ASS1-KD
NS.
7
14
21
28
(Days)
Tumor volume (cm3)
3
2
1
0
Control
PYCR1-KD
NS.
7
14
21
28
(Days)

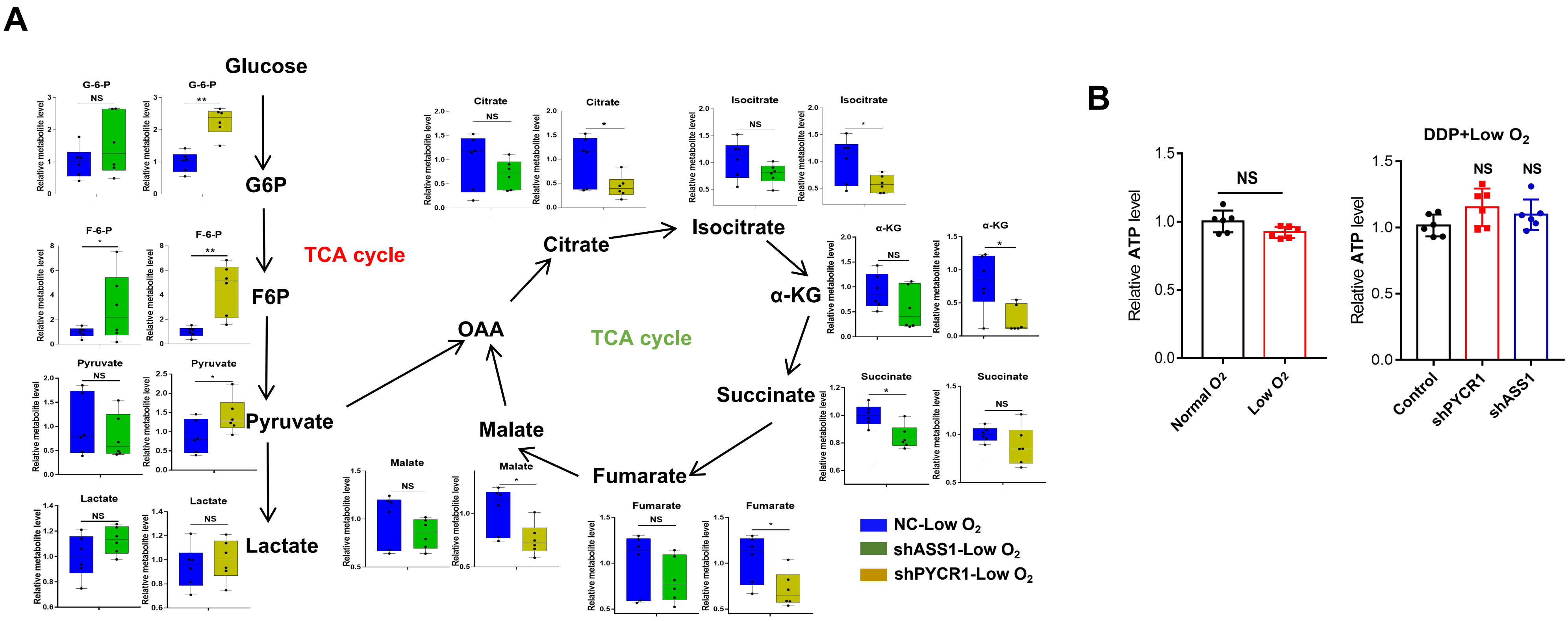

A
Glucose
G6P
F6P
Pyruvate
Lactate
TCA cycle
OAA
Citrate
Isocitrate
α-KG
Succinate
Fumarate
Malate
TCA cycle
G-6-P
F-6-P
Pyruvate
Lactate
Citrate
Isocitrate
α-KG
Succinate
Fumarate
Malate
Relative metabolite level
NS
*
**
NC-Low O2
shASS1-Low O2
shPYCR1-Low O2
B
Relative ATP level
Normal O2
Low O2
NS
DDP+Low O2
Control
shPYCR1
shASS1
