# Supplementary material for: Targeting IGF1R signaling enhances the sensitivity of cisplatin by inhibiting proline and arginine metabolism in oesophageal squamous cell carcinoma under hypoxia
Source: J Exp Clin Cancer Res. 2023 Mar 28;42:73. doi: 10.1186/s13046-023-02623-2 (PMC10044411; doi:10.1186/s13046-023-02623-2)
Supplement: Supplementary file 2 — Additional file 2: Table S1. Primers for real-time PCR assays. [file 13046_2023_2623_MOESM2_ESM.docx]

**Supplementary Table 1**

Primers for real-time PCR assays.

| IGF1R | forward | 5'-GTTGGTGATTATGCTGTACGTC-3' |
| --- | --- | --- |
|  | reverse | 5'-TCCTTCATAGACCATCCCAAAC-3' |
| ASS1 | forward | 5'-CTTCATGTACCTGAACGAAGTC-3' |
|  | reverse | 5'-TCGATGTCTAAATGAGCATGGT-3' |
| PYCR1 | forward | 5'-TCCATTGAGAAGAAGCTGTCAG-3' |
|  | reverse | 5'-CATCAATCAGGTCCTCTTCCAC-3' |
| C-MYC | forward | 5'-AAGTCCTGCGCCTCGCAA-3' |
|  | reverse | 5'-GCTGTGGCCTCCAGCAGA-3' |
| ASS1 primer 1  (for ChIP) | forward | 5'-GCTGTGAACGCTGAGCGGCTCCA-3' |
|  | reverse | 5'-CCTCCTCCTCTGGGCCCGGGAAG-3' |
| ASS1 primer 2  (for ChIP) | forward | 5'-TGCATCATTTTCCCAAGCCCATC-3' |
|  | reverse | 5'-GTGAAGCAATGCATGGACCTGGC-3' |
| PYCR1(for ChIP) | forward | 5'-CCAGAGCGGTTGCATCATCTGCG-3' |
|  | reverse | 5'-CGTGTCCGGGTGGCCCAAGACAA-3' |
