# Supplementary material for: Targeting IGF1R signaling enhances the sensitivity of cisplatin by inhibiting proline and arginine metabolism in oesophageal squamous cell carcinoma under hypoxia
Source: J Exp Clin Cancer Res. 2023 Mar 28;42:73. doi: 10.1186/s13046-023-02623-2 (PMC10044411; doi:10.1186/s13046-023-02623-2)
Supplement: Supplementary file 3 — Additional file 3: Table S2. Association of prognosis with clinical characteristics. [file 13046_2023_2623_MOESM3_ESM.docx]

**Supplementary Table 2** Association of prognosis with clinical characteristics.

| Clinical characteristics | Prognosis | | |
| --- | --- | --- | --- |
|  | Death | Survival | *P* value**^a^** |
| **Gender** |  |  |  |
| Male | 55 | 10 | 0.691 |
| Female | 11 | 3 |  |
| **Age, years** |  |  |  |
| ≤60 | 19 | 8 | **0.023** |
| ＞60 | 47 | 5 |  |
| **Tumor size** |  |  |  |
| ≤3cm | 28 | 9 | 0.127 |
| ＞3cm | 38 | 4 |  |
| **Metastasis** |  |  |  |
| Negtive | 46 | 12 | **0.030** |
| Positive | 20 | 1 |  |
| **Clinical stage** |  |  |  |
| 0-II | 54 | 11 | 0.809 |
| III-IV | 12 | 2 |  |
| **IGF1R expression** | 2.25 ± 0.12 | 1.73 ± 0.17 | **0.045** |

**^a^**Statistical significance is determined by the Chi-square test, Fisher’s exact test or Student’s *t* test.
