# Supplementary material for: Targeting IGF1R signaling enhances the sensitivity of cisplatin by inhibiting proline and arginine metabolism in oesophageal squamous cell carcinoma under hypoxia
Source: J Exp Clin Cancer Res. 2023 Mar 28;42:73. doi: 10.1186/s13046-023-02623-2 (PMC10044411; doi:10.1186/s13046-023-02623-2)
Supplement: Supplementary file 4 — Additional file 4: Table S3. Summary of the carnitine metabolism. [file 13046_2023_2623_MOESM4_ESM.docx]

**Supplementary Table 3** Summary of the carnitine metabolism.

| Abbreviation | Common name | Category**^a^** | Formula | Trend |
| --- | --- | --- | --- | --- |
| C0 | L-carnitin | SSC | C_7_H_15_NO_3_ | NS |
| C2 | Acetyl-L-carnitine | SSC | C_9_H_17_NO_4_ | NS |
| C3 | Propionyl-L-carnitine | SSC | C_10_H_19_NO_4_ | ↓* |
| C4 | Butyryl-L-carnitine | SSC | C_11_H_21_NO_4_ | ↓** |
| C5 | Valeryl-L-carnitine | SSC | C_12_H_23_NO_4_ | ↓** |
| C6 | Hexanoyl-L-carnitine | SSC | C_13_H_25_NO_4_ | ↓** |
| C8 | Octanoyl-L-carnitine | SSC | C_15_H_29_NO_4_ | ↓** |
| C12 | Dodecanoyl-L-carnitine | SSC | C_19_H_37_NO_4_ | ↓* |
| C14 | Tetradecanoyl-L-carnitine | SSC | C_21_H_41_NO_4_ | ↓* |
| C16 | Hexadecanoyl-L-carnitine | SSC | C_23_H_45_NO_4_ | ↓* |
| C18 | Octadecanoyl-L-carnitine | SSC | C_25_H_49_NO_4_ | NS |
| C5OH | Hydroxyvalery-L-carnitine | HSC | C_12_H_25_NO_5_ | ↓* |
| C10:1 | Decenoyl-L-carnitine | SMC | C_17_H_31_NO_4_ | NS |
| C16:1 | Hexadecenoyl-L-carnitine | SMC | C_23_H_43_NO_4_ | ↓** |
| C18:1 | Octadecenoyl-L-carnitine | SMC | C_25_H_47_NO_4_ | NS |
| C6DC | Methylglutaryl-L-carnitine | DCC | C_13_H_23_NO_6_ | ↓* |

**P*<0.05; ***P*<0.01; ↑, upregulated; ↓, downregulated

**^a^**DCCs, di-carboxylic acylcarnitines; HSCs, hydroxyl saturated acylcarnitines; SMCs, straight mono-unsaturated acylcarnitines; SSCs, straight saturated acylcarnitines.
